# Predictors of functional outcome in patients with major depression and bipolar disorder: A dynamic network approach to identify distinct patterns of interacting symptoms

**DOI:** 10.1371/journal.pone.0276822

**Published:** 2023-02-15

**Authors:** Giuseppe Alessio Platania, Claudia Savia Guerrera, Pierfrancesco Sarti, Simone Varrasi, Concetta Pirrone, Dina Popovic, Andrea Ventimiglia, Simona De Vivo, Rita Anna Cantarella, Fabio Tascedda, Filippo Drago, Santo Di Nuovo, Chiara Colliva, Filippo Caraci, Sabrina Castellano, Johanna M. C. Blom

**Affiliations:** 1 Department of Educational Sciences, University of Catania, Catania, Italy; 2 Department of Biomedical and Biotechnological Sciences, University of Catania, Catania, Italy; 3 Department of Biomedical, Metabolic and Neural Sciences—University of Modena and Reggio Emilia, Modena (MO), Italy; 4 Abarbanel Mental Health Center, Bat-Yam, Israel; 5 Villa dei Gerani Clinic ASP3 Catania, Catania, Italy; 6 Department of Mental Health, ASP3 Catania, Catania, Italy; 7 Department of Life Sciences, University of Modena and Reggio Emilia, Modena, Italy; 8 Center for Neuroscience and Neurotechnology, University of Modena and Reggio Emilia, Modena, Italy; 9 Azienda Unità Sanitaria Locale di Modena, Distretto di Carpi, Modena, Italy; 10 Department of Drug and Health Sciences, University of Catania, Catania, Italy; 11 Oasi Research Institute—IRCCS, Troina, Italy; National Institutes of Health, UNITED STATES

## Abstract

The purpose of this study is to use a dynamic network approach as an innovative way to identify distinct patterns of interacting symptoms in patients with Major Depressive Disorder (MDD) and patients with Bipolar Type I Disorder (BD). More precisely, the hypothesis will be testing that the phenotype of patients is driven by disease specific connectivity and interdependencies among various domains of functioning even in the presence of underlying common mechanisms. In a prospective observational cohort study, hundred-forty-three patients were recruited at the Psychiatric Clinic “Villa dei Gerani” (Catania, Italy), 87 patients with MDD and 56 with BD with a depressive episode. Two nested sub-groups were treated for a twelve-week period, which allowed us to explore differences in the pattern of symptom distribution (central vs. peripheral) and their connectedness (strong vs weak) before (T0) and after (T1) treatment. All patients underwent a complete neuropsychological evaluation at baseline (T0) and at T1. A network structure was computed for MDD and BD patients at T0 and T1 from a covariance matrix of 17 items belonging to three domains–neurocognitive, psychosocial, and mood-related (affective) to identify what symptoms were driving the networks. Clinically relevant differences were observed between MDD and BD, at T0 and after 12 weeks of pharmacological treatment. At time T0, MDD patients displayed an affective domain strongly connected with the nodes of psychosocial functioning, while direct connectivity of the affective domain with the neurocognitive cluster was absent. The network of patients with BD, in contrast, revealed a cluster of highly interconnected psychosocial nodes but was guided by neurocognitive functions. The nodes related to the affective domain in MDD are less connected and placed in the periphery of the networks, whereas in BD they are more connected with psychosocial and neurocognitive nodes. Noteworthy is that, from T0 to T1 the “Betweenness” centrality measure was lower in both disorders which means that fewer “shortest paths” between nodes pass through the affective domain. Moreover, fewer edges were connected directly with the nodes in this domain. In MDD patients, pharmacological treatment primarily affected executive functions which seem to improve with treatment. In contrast, in patients with BD, treatment resulted in improvement of overall connectivity and centrality of the affective domain, which seems then to affect and direct the overall network. Though different network structures were observed for MDD and BD patients, data suggest that treatment should include tailored cognitive therapy, because improvement in this central domain appeared to be fundamental for better outcomes in other domains. In sum, the advantage of network analysis is that it helps to predict the trajectory of future phenotype related disease manifestations. In turn, this allows new insights in how to balance therapeutic interventions, involving different fields of function and combining pharmacological and non-pharmacological treatment modalities.

## Introduction

Major Depressive Disorder (MDD) and BipolarDisorder (BD) are among the most debilitating and prevalent mental disorders, often leading to substantial functional impairment [1–3]. Patients with MDD or BD represent an enormously heterogenous group while identified risk factors lack the capacity to distinguish individual trajectories. Also, no single treatment modality has proved to be truly effective in preventing relapse or the worsening of symptoms. At present, research indicates that the profoundly engrained traditional categorical approach linking just one or a few individual mediators (often molecular or biological) to an illness related phenotype, has provided limited understanding of the complex underlying pathologic conditions. More importantly, this traditional approach has often hampered progress in the development of personalized and more efficacious treatments.

Consequently, a paradigm shift is necessary that invests in the detection of trajectories of disease which allow to better understand the specific evolution of the clinical pattern of different patient populations over time. Innovative discoveries from other fields, such as, imaging techniques and mathematical modeling together with graph analysis have led to new conceptual thinking resulting in increasingly explanatory and predictive models which may offer a more realistic image representation of the psychosocial strengths and needs of patients. A dynamic network approach, able to model interacting neurocognitive, psychosocial, and mood-related determinants of MDD and BD, represents such an approach and allows to explain the individual behavioral variances of different patient groups. Based on a multimodal approach [4], it gathers evidence from different realms of function such as subtle neurocognitive disfunction and uses them (together with biomarkers) as clinical predictors of risk [5].

The network analysis model is of growing interest in the study of contemporary psychopathology. Network analysis studies the relationships between symptoms and their triggers [6] and has challenged the traditional latent disease approach [7, 8]. Recent studies highlight that many clinically meaningful findings emerge from the study of the correlations between symptoms using a network approach [9, 10] and suggest the presence of underlying relationships common to multiple psychiatric disorders but different at the phenotypic level. In fact, several studies have introduced network analysis and found that different cognitive, emotional, and psychosocial patterns are present in different psychopathological conditions, accentuating that patients’ characteristics are not simply the sum of separate abilities but the result of complex dynamic interactions [8, 11].

Based on these premises, here we used network analysis to study the fine-grained phenotypes of MDD and BD hypothesizing that the integration of data across diverse levels of analysis will capture the nature of their dynamic relationship over time, both among patients and between treatment modalities. Furthermore, using network analysis and graph theory, we tested the hypothesis that patients with MDD and BD display different connections among symptoms, which may change, strengthen, sustain, or weaken each other over time [12].

Also, the influence of symptoms on the development of other symptoms might not be the same or distributed equally in the two pathologies. What is ultimately important, is the pattern of connections between symptoms in relation to the functional impairment observed in each pathology. Additionally, network analysis will help to understand what factors are driving the network and what are the differences and similarities between the driving factors. Lastly, this type of analysis may provide new insights regarding the choice of pharmacological treatment to more effectively treat MDD and BD [10, 13].

Few studies have used this methodology to study affective disorders, especially when comparing unipolar and bipolar Depression [14]. Galimberti and colleagues [15] conducted a study using network analysis to examine possible differences between MDD and BD from a cognitive perspective. Results showed that the BD network was less connected when compared to the MDD network. Also, in BD, executive dysfunction was more central, while in MDD, memory impairment played a key role with a strong impact on functional impairment [15].

Given that unipolar and bipolar Depression are often misdiagnosed [16], network analysis could represent a new and useful tool and improve both the diagnosis and treatment of these diverse affective disorders. The dynamic organization of different functions in networks of interdependent factors Willemstad underscore the differences between unipolar and bipolar Depression and, thus, enhance the accuracy of the differential diagnosis. Moreover, network analysis provides an important tool to verify the impact of treatment by monitoring changes in interdependencies as well as the configuration of symptoms and their connections and development over time.

A large body of evidence demonstrates that MDD and BD display mainly cognitive deficits, affective symptoms, and psychosocial impairment. In MDD, for instance, cognitive deficits consist of executive dysfunction, verbal and visual memory impairment, reduction of motor speed and attention, which persist long after affective symptoms have subsided [17–19]. Also, baseline cognitive performance still influenced the performance of subjects one-year into follow-up. In a recent study, Castellano et al. demonstrated a critical role for verbal memory in relation to psychosocial functioning after one year of treatment in MDD patients, partially responding to treatment [20].

Diversely, in BD, cognitive deficits influenced social and professional skills, with an overall negative impact on quality of life [19, 21], episodic memory [22], attention [23], fine motor skills [24], reduced psychosocial functioning (negatively affected by memory and depressed mood [25], and executive functions [26, 27]). Psychosocial health of BD patients was also impaired by psychomotor agitation, irritability, insomnia, and emotional lability [19, 28, 29].

In light of this, network analysis provides a way to visualize and understand how cognitive, affective, and psychosocial symptoms and capacities interact with and depend on each other before and after treatment with psychotropic drugs. While a psychotherapeutic approach helps patients to manage the cognitive biases underlying their way of thinking [30] and often effectively reduces the severity of symptoms, pharmacological treatment improves cognitive and affective symptoms [31]. Recently, a prospective observational study was conducted on the effectiveness of SSRIs and SNRIs in a sample of 33 MDD patients. Cognitive and affective assessment, performed at baseline and at 4 and 12 weeks into treatment, showed that SSRIs and SNRIs improved cognitive symptoms in MDD independently of their efficacy on affective symptoms [32].

Ultimately, the purpose of our study is to use network analysis to clarify the structure of the relationships between affective, cognitive, and psychosocial symptoms and capacities in a sample of 87 MDD and 56 BD patients. Moreover, two nested sub-groups of patients underwent a twelve-week period of pharmacological treatment, allowing to explore differences in the pattern of symptom distribution (central vs. peripheral) and their connectedness (strong vs weak) before (T0) and after (T1) treatment. Finally, the overall aim is to better understand the interaction between neurocognition, psychosocial functioning and affective symptoms, to identify which symptoms are driving the network of patients with MDD compared to BD and consequently guide the implementation of individualized effective treatment plans aimed to promote functional recovery.

## Material and methods

### Subjects

Patients were recruited at the Psychiatric Clinic “Villa dei Gerani” (Catania, Italy). All patients received oral and written information on the planned use of the data and provided written informed consent. The study was conducted in accordance with the Declaration of Helsinki.

The study was approved by the ethical committee of the “Azienda Sanitaria Provinciale 3 (ASP3) of Catania of which the “Villa dei Gerani Clinic” (clinical coordinator of the study), is part (Approval date of the extended study July 24, 2012). The study met the ethical administrative requirements under Italian legislation in force when the study’s administrative process started (03.06.2012) according to CM 6 02.09.2002, GU 214 12.09.2002 and D 29.03.2008 of the Italian Medicine Agency (Agenzia Italiana del Farmaco, AIFA) and GU 76 31.03.2008, Art 10 (Procedures for Observational Studies).

The study design was a prospective, observational (non-interventional), cohort study. The study complied with the definition of “observational” study (i.e., “non-interventional”) provided in Article 2(c) of Directive 2001/20/EC, meaning that the investigator who carries out the study does not interfere with the physician’s decision regarding which drug is to be prescribed to each individual patient. Therefore, prescription of antidepressants, antipsychotics, or mood stabilizers resulted solely from an independent clinical evaluation, according to the physician’s clinical judgment, and based on each patient’s clinical profile (presence of a depressive episode). Moreover, the decision to include a patient in the study, following his/her consent, was taken independently of the clinical decision to prescribe psychotropic drugs. Finally, the study did not affect the medical practice of participating physicians and did not trigger additional medical visits.

One hundred-forty-three patients (97 females and 46 males, mean age 50.78 ± 10.18) were recruited for this study, and 45 of them (20 males and 25 females, mean age 50.68 ± 10.25) completed the 12 weeks of treatment (Table 1). Among the 143 patients at T0, 87 were diagnosed with Major Depressive Disorder (29 males, 58 females, mean age 52.38), and 56 with Bipolar I Disorder with a depressive episode (19 males, 37 females, mean age 53). Among the 45 patients at T1, who completed the study after 12 weeks of treatment, 16 were diagnosed with Major Depressive Disorder (5 males, 11 females, mean age 53.62) and 29 with Bipolar I Disorder with a depressive episode (12 males, 17 females, mean age 50.86).

**Table 1:** Demographic and clinical characteristics of the studied population with their relative percentages.

ALL			Major Depressive Disorder		Bipolar Disorder	
**DEMOGRAPHICS**	**SAMPLE**	**PERCENTAGE**	**SAMPLE**	**PERCENTAGE**	**SAMPLE**	**PERCENTAGE**
Sample size	143	100	87	100	56	100
**Gender**			**Gender**			
Male	46	29.9	29	33.3	17	30.4
Female	97	70.1	58	66.7	39	69.6
Mean Age	50.78	\	52.38	\	53	\
**Marital status**			**Marital status**			
Unmarried	34	24.3	18	20.7	16	28.6
Married	68	47.2	45	51.7	23	41.1
Divorced	29	20.1	17	19.5	12	21.4
Widow	12	8.3	7	8.0	5	8.9
**Education**			**Education**			
Primary school	13	9	11	12.6	2	3.6
Secondary school	54	37.5	34	39.1	20	35.7
High school	58	40.4	29	33.3	29	51.8
University and more	18	13.1	13	14.9	5	8.9
**Employment status**			**Employment status**			
Student	1	0.7	0	0.0	1	1.8
Employed	87	60.4	51	58.6	36	64.3
unemployed/Retired/Housewife	55	38.9	36	41.4	19	33.9
**Age at onset**			**Age at onset**			
Before 20 years old	33	22.9	20	23.0	13	23.2
After 20 years included	110	77.1	67	77.0	43	76.8
**Previous depressive episodes**			**Previous depressive episodes**			
0	11	7.6	4	4.6	7	12.5
1	57	39.6	39	44.8	18	32.1
2	64	45.2	38	43.7	26	46.4
3	11	7.6	6	6.9	5	8.9

The criteria for inclusion in the study were

1) A diagnosis of MDD or BD (Type I) according to DSM-V criteria.

2) Age between 18–65 years old.

Criteria for exclusion from the study were

1) A history of mental retardation or any clinical condition that could affect cognitive performance.

2) Axis I comorbidity.

3) Electroconvulsive therapy 1 year prior to neuropsychological assessment.

### Pharmacological treatment

Between T0 (first neuropsychological evaluation) and T1 (second evaluation) forty-five [33] patients followed a twelve-week treatment tailored to the needs of the individual patient. Treatments can be summarized as follows:

1. Sixteen patients with Major Depressive Disorder were treated exclusively with ANTIDEPRESSIVE drugs (SSRIs, SNRIs and tricyclics).
2. Eight of the patients with Type I Bipolar Disorder were given GENERATION I and II ANTIPSICOTICS (Treatment 1).
3. The remaining twenty-one patients with Bipolar Disorder Type I were treated with GENERATION II ANTIPSICOTICS and MOOD STABILIZERS (Treatment 2).

In terms of constructing the networks, the two treatment subgroups of patients with bipolar I disorder were combined which allowed us to make observations about the change itself and not the specific treatment.

### Neuropsychological assessment

Patients underwent a complete neuropsychological evaluation carried out at baseline and at the end of 12-weeks of pharmacological treatment. At baseline depressive symptoms were assessed by the Hamilton Depression Rating Scale (HDRS) and the Beck Depression Inventory (BDI–II). Patients were also assessed using a comprehensive neuropsychological battery consisting of: 1) Tools for the assessment of global cognitive function: Mini Mental State Examination (MMSE) and Montreal Cognitive Assessment (MoCA), and 2) Tools for the assessment of specific cognitive functions: Rey 15 Words Test and Verbal Memory Span (Digit Span), the Phonetic Verbal Fluency test (FAS), the “Vocabulary” test from the WAIS-IV, and finally, the Frontal Assessment Battery to measure executive functions (FAB). More specifically, with executive functions we intend functions controlled primarily by the frontal lobes like short-term memory, working memory, planning, inhibition, and attention in all its declination (sustained, alternating, and divided). All these functions were explored with some subtests contained in the MoCA, MMSE, and FAB (all considered neuropsychological tests for global screening of cognitive functions) and more specific tests:

-     SPAN-A (forward) for short verbal memory
-     SPAN-I (backward) for working memory
-     REY (15 Rey’s words–Immediate recall) for short-term verbal memory for unstructured material
- FAS (Test of Phonemic Fluency) for evaluating vocabulary and lexical organization on phonemic cue (this means not accessing the semantic level). In this test people have to produce words not accessing the semantic warehouse and, at the same time, trying not to say words already said, proper names and with the same root. This means using inhibition, short-term memory, planning, and self-monitoring; all executive functions (frontal).

In addition, FAB also contains a subtest of Phonemic Fluency giving the patient the letter “S” and 1 minute to say all the words that start with that letter.

In the article we talked about executive functions because FAS also investigate them.

### Functional assessment

The Functioning Assessment Short Test (FAST) 25 was used as a primary outcome of psychosocial risk at the study endpoints to identify predictors for specific domains of function, such as: autonomy (Atn), occupational functioning (Occ), cognitive functioning (Cog), financial issues (Fnn), interpersonal relationships (Int), and leisure time (Lsr). The FAST was assessed after 12 weeks from the start of the pharmacological treatment. For this study, we only included the score of the overall functioning and the score of six specific FAST scale domains.

### Statistical analyses

A network structure was computed for MDD and type I bipolar adult patients at the onset of the study (T0) and after twelve weeks of treatment (T1). A covariance matrix of 17 items belonging to three domains–the neurocognitive, social, and mood-related domains–was used to analyze the interaction between neurocognition, psychosocial functioning and affective symptoms, and to identify what symptoms were driving the network and could be targets for effective treatment plans to promote functional recovery.

The structure of a network model is characterized by two main elements: nodes and edges. Usually, nodes are depicted with circles and represent, in the psychopathological scenario, the symptoms of a disorder [11]; the edges are depicted with lines connecting nodes to each other, and represent, the relationships between symptoms [12, 34].

Generally, three main measures of centrality are considered: strength/degree, betweenness and closeness centrality.

- **DEGREE CENTRALITY (STRENGTH/DEGREE):** the number of connections of a node: the more connections it has, the more important it is.
  Clinically speaking, if a symptom (e.g., depressed mood) has many connections within a psychopathological system, it may be considered as a risk factor for the development of a variety of other symptoms. If, on the other hand, it has a low number of connections, it is considered peripheral with a scarce risk of fostering or influencing other symptoms [35]. The strength of a node in a weighted network is given by the product of the number of nodes to which a node is connected and the average of the weights of these nodes, adjusted for a tuning parameter:
$$C_{D}^{w\alpha }(i)=k_{i}\times {\left(\frac{s_{i}}{k_{i}}\right)}^{\alpha }=k_{i}^{\left(1-\alpha \right)}\times s_{i}^{\alpha }$$
  where **α** is the positive tuning parameter that is chosen based on the data and **ki** and si are the Degree and Strength of the nodes, respectively:
$$k_{i}=C_{D}(i)=\overset{N}{\underset{j}{\sum }}x_{ij}\;s_{i}=C_{D}^{w}(i)=\overset{N}{\underset{j}{\sum }}w_{ij}$$
  where **i** is the reference node, **j** are all other nodes, **N** is the total number of nodes, and **x** is the adjacency matrix in which the cell **xij** is defined 1 if node **i** is connected to node **j**, and 0 otherwise. **W** is the weighted adjacency matrix.
- **BETWEENNESS:** measures how a node is involved in the shortest path between other nodes [15]. It is used to determine which nodes are most likely to connect other nodes to each other and, therefore, which are most likely to facilitate connections in the network. For example, through this measure it is possible to determine the most important doins affecting the connectivity between problems and symptoms [36]. The algorithm for calculating "shortest paths" is that of Dijkstra (1959) [37], implemented in R and repurposed by Opsahl, Agneessens and Skvoretz (2010) [38]. The length of the shortest path between two nodes is defined as follows:
$$d^{w\alpha }(i,j)=\min(\frac{1}{{\left(w_{ih}\right)}^{\alpha }}+\ldots +\frac{1}{{\left(w_{hj}\right)}^{\alpha }})$$
  This algorithm can be used directly for the Closeness measure (described below) and considers both the number of intermediate nodes and the weight of connections.
  The combination of the formula of Freeman (1978) [39] and the above formula of Dijkstra leads to a final formulation of the Betweenness parameter formula:
$$C_{B}^{w\alpha }(i)=\frac{g_{jk}^{w\alpha }(i)}{g_{jk}^{w\alpha }}$$
  Where **gjk** is the number of shortest paths between two nodes, **gjk(i)** is the number of those paths passing through node i, and **α** is the positive tuning parameter.
- **CLOSENESS:** is used to understand the importance of a symptom and its immediate impact on neighboring symptoms or functions. The nodes with the highest closeness quickly affect other nodes and, in turn, they are more likely to be influenced.

The combination of these three different measures, strength/degree, betweenness, and closeness identifies the domains that are most important in the configuration of a network and improves our understanding regarding the symptoms to target with precise individualized therapeutic interventions.

Network construction was based on Spearman correlations by defining negative/positive relationships between nodes, so that weighted undirected networks could be built. The thickness and color of a connection represent the strength and the sign–positive (in black) or negative (in red)–of significant correlations (p<0.05): the more accentuated the line, the stronger the association. In the absence of an edge, the relationship was 0. The correlations were assessed through various psychometric tools: HDRS and BDI-II for affective symptoms, FAST for psychosocial functioning, MoCA and MMSE for global neurocognition, FAS (phonetic verbal fluency), Vocabulary, backward/forward Span, Rey Memory Test and FAB for specific cognitive functions.

The resulting networks were analyzed considering three measures of centrality: Strength, Betweenness and Closeness. Networks were estimated using the package “qgraph” in the R software by the Fruchterman-Reingold algorithm which is based on an iterative procedure that places the most crucial nodes in the center of the network, whereas the weakest nodes are placed in the periphery. This algorithm is automatically calculated using the command “layout = spring”. The Fruchterman & Reingold (FR) algorithm transforms the network into a system of particles with mass. The nodes are interpreted as particles, and the edges as the pushes they give each other through attractive forces (calculated between adjacent vertices) and repulsive forces (between pairs of vertices). Furthermore, to reduce the quadratic complexity of the repulsive forces, the algorithm ignores these forces between distant vertices.

Also, we added another feature to the networks: a predictability ring around each node using the package “mgm” in R software. It shows the degree to which a given node can be predicted by all other nodes in the network with which it has connections. Predictability is an important measure when considering psychopathology because it tells us on an interpretable absolute scale how much a node is determined by other nodes in the network allowing for increasingly explanatory parameters of risk (importance of a node). Because this measure gives us an idea of how clinically relevant connections are, it is useful to estimate the potential success of clinical interventions which could thereby effectively guide treatment selection. As predictability measures, we selected, for continuous variables, the root mean square error (RMSE) as a p*roportion of the explained variance* [40].

The labels of each node are summarized as follows.

Neurocognitive domain (pink color in the images): MCA = Montreal Cognitive Assessment, MMS = Mini Mental State Examination, FAB = Frontal Assessment Battery, FAS = Phonetic verbal Fluency, Vcb = Vocabulary, REY = Rey 15 Words immediate recall, RDC = Rey 15 Words deferred recall, SPAN_A = digit span forward, SPAN_I = digit span backward.

Depression related domain (blue color in the images): BDI = Beck depression inventory II, HDR = Hamilton depression rating scale.

Psychosocial domain (green color in the images): Cgn = Cognitive functioning, Occ = occupational functioning, Atn = Autonomy, Lsr = Leisure time, Int = interpersonal relationships, Fnn = financial issues.

## Results

### Differences between major depressive disorder and type I bipolar depression at the onset of the study (Time 0)

**Fig 1:**
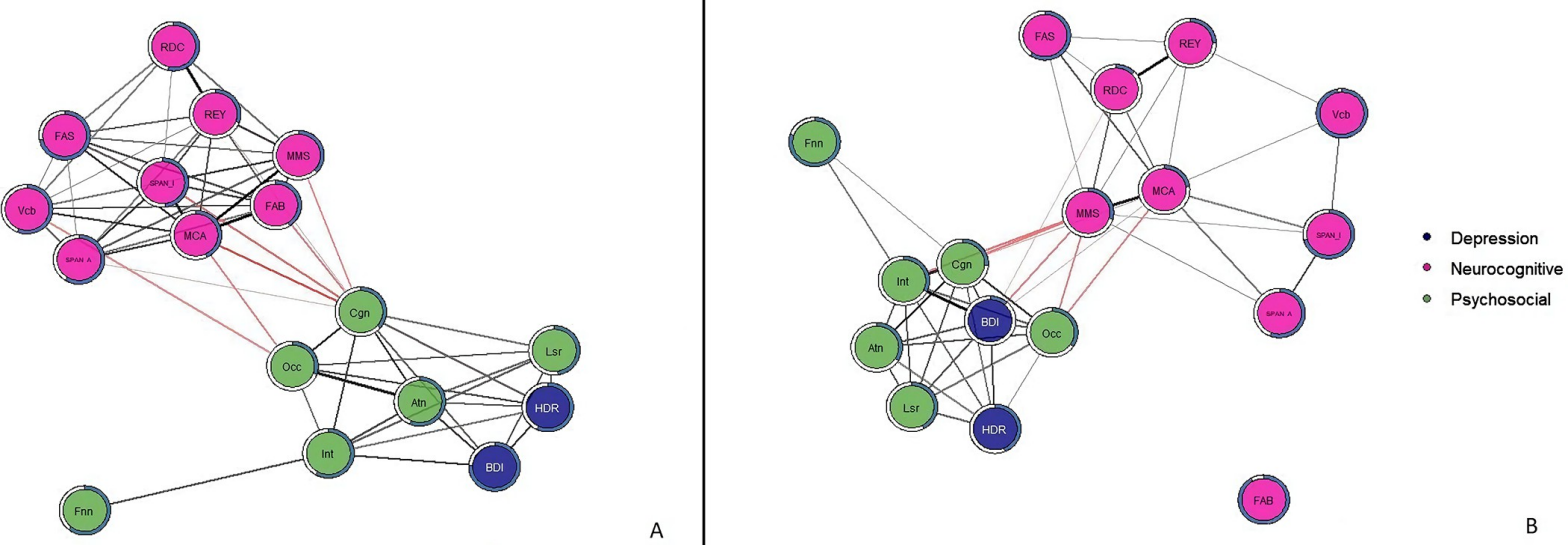
Resulting networks at the onset of the study (T0). Part A represents the network of patients with MDD. Part B represents the network of Type I BD patients. Pink color groups the neurocognitive domain, green color the psychosocial domain and blue color the depression-related nodes. Black edges represent positive Spearman correlations, red edges negative Spearman correlations. The blue ring around each node represents its predictability and the fuller the bar is, the higher the RMSE value is. Cognitive domain: MoCA = Montreal Cognitive Assessment, MMS = Mini Mental State Examination, FAB = Frontal Assessment Battery, FAS = Phonetic verbal Fluency, Vcb = Vocabulary, REY = Rey 15 Words immediate recall, RDC = Rey 15 Words deferred recall, SPAN_A = digit span forward, SPAN_I = digit span backward. Depression related domain (blue color in the images): BDI = Beck depression inventory II, HDR = Hamilton depression rating scale. Psychosocial domain (green color in the images): Cgn = Cognitive functioning, Occ = occupational functioning, Atn = Autonomy, Lsr = Leisure time, Int = interpersonal relationships, Fnn = financial issues.

**Fig 2:**
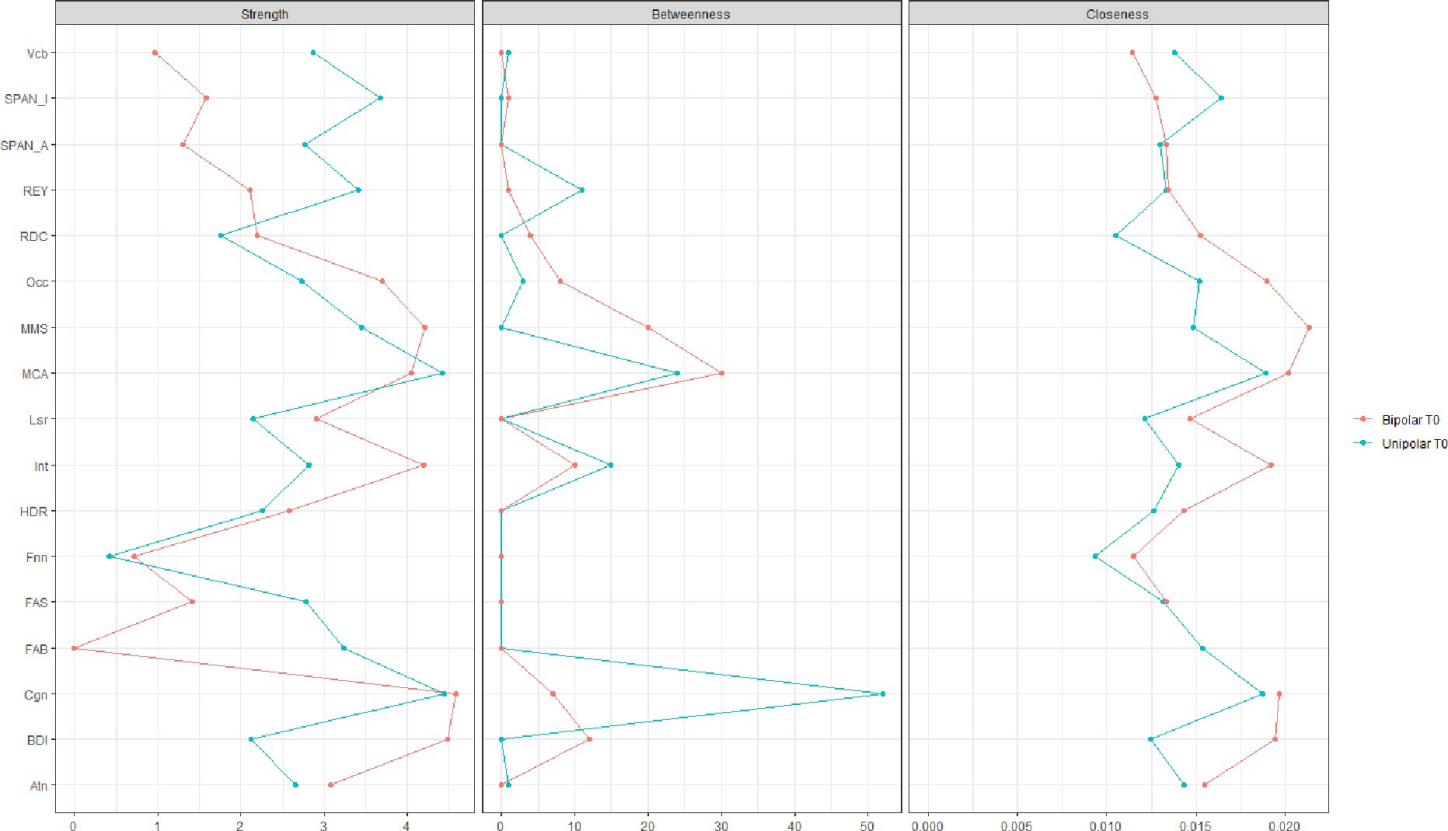
Centrality measures (strength, betweenness, closeness) of MDD and BD patients at baseline (T0). Red color represents Bipolar Patients and Blue Color MDD patients. MoCA = Montreal Cognitive Assessment, MMS = Mini Mental State Examination, FAB = Frontal Assessment Battery, FAS = Phonetic verbal Fluency, Vcb = Vocabulary, REY = Rey 15 Words immediate recall, RDC = Rey 15 Words deferred recall, SPAN_A = digit span forward, SPAN_I = digit span backward, BDI = Beck depression inventory II, HDR = Hamilton depression rating scale, Cgn = Cognitive functioning, Occ = occupational functioning, Atn = Autonomy, Lsr = Leisure time, Int = interpersonal relationships, Fnn = financial issues.

As shown in Fig 1A, the affective cluster in patients with MDD is placed in the periphery and the two nodes represented by HDRS and BDI do not occupy a central role in driving the network. Although the affective domain is highly connected with the nodes of psychosocial functioning, direct connectivity with the neurocognitive cluster is absent. The central domain of affective appraisal served as a bridge between the psychosocial domain and the neurocognitive domain. No direct connectivity was observed among specific frontal related executive functions and the psychosocial domain. With frontal related functions we intend functions controlled primarily by the frontal lobes like short-term memory, working memory, planning, inhibition, and attention in all its declination (sustained, alternating, and divided). Moreover, psychosocial ability and depressive symptoms seem to form one separate system. while frontal related functions compose another world. The latter is represented by diffusely connected nodes, with MoCA and MMSE driving the neurocognitive cluster, assuming a bridge function to the more emotional psychosocial area (Figs 1 and 2). In fact, the majority of connections start from the MoCA and MMSE nodes and go to the Psychosocial and Affective nodes. In addition, Centrality measures show that these two nodes, of all others, have higher values of “Betweenness”. This means that many "shortest paths" go through the MoCA and MMSE nodes. In addition, clusters are internally hyperconnected, demonstrating low resilience to change induced by external positive or negative factors [41].

The network of Bipolar I patients at T0 is characterized by a main division in two dimensions–a neurocognitive and a psychosocial one–and displays a different pattern of connectivity among nodes (Fig 1B). The cluster of neurocognition is scarcely connected and heavily driven by functions tested with the MoCA (Fig 2). Furthermore, the Affective nodes of patients with BD are diffusely related to the domain of psychosocial functioning. Psychosocial impairment especially related to interpersonal (Int) and occupational (Occ). Interpersonal (Int) and Occupational (Occ) nodes drive the area of psychosocial functioning because they have the highest “Betweenness” and “Strength” values (centrality measures) and are only second to the Cognitive (Cgn) node. Now because this last node is the one that has the majority of edges connecting the psychosocial and neurocognitive clusters, its centrality measures are affected by the dual influence of these two domains. In addition, the “Cognitive” node has much more in common with the neurocognitive node than “Int” and “Occ”. For these reasons, “Int” and “Occ” are more segregated and better reflect the influence of the psychosocial domain.

The network of patients with MDD displayed a central role for cognitive-emotional control which proved highly significant in driving the psychosocial and depression-related clusters of symptoms. Cognitive-emotional control provides a bridge to the neurocognitive cluster, as evidenced by an elevated level of betweenness centrality (Fig 2). In contrast, in patients with BD the executive function cluster was the most influential and likely drives the neurocognitive domain overall. Though the psychosocial domain is characterized by substantial closeness centrality, only the nodes “Cognitive” (Cgn) and “Interpersonal” (Int) display the highest number of connections in this cluster.

Predictability measures expose an additional difference between unipolar and bipolar depression. Values tend to be higher in unipolar depression than in bipolar depression; this means that nodes in the unipolar network "better" explain the variance among themselves than from external factors. In contrast, the backbone of bipolar disorder is a higher genetic imprint that determines aspects of functional brain organization. This is further supported by the fact that in the BD network the nodes representing frontal functions are placed in the outer part of the neurocognitive cluster (Fig 1B).

In sum, at the onset of the study (baseline), the network of MDD patients is driven by three well connected clusters, with cognitive-emotional ability or disability driving the psychosocial domain and providing a bridge connecting depression-related symptoms to the neurocognitive cluster. The network of depressed patients with type I bipolar disorder, on the other hand, is characterized by a highly interconnected cluster of psychosocial nodes but driven by neurocognitive functions. Neurocognitive functions in BD are less connected among each other (fewer edges among the nodes) than in MDD where the neurocognitive world displays a number of edges connecting nodes among each other. Noteworthy, in patients with MDD, the indexes related to the affective domain are less connected and placed in the periphery of the network, whereas in BD patients they are more connected with psychosocial and neurocognitive nodes. Lastly, predictability measures show additional differences between unipolar and bipolar depression and could be used to better understand the relationships between symptomatology and the functions investigated.

### Differences between MDD and BD after twelve weeks of treatment (Time 1)

**Fig 3:**
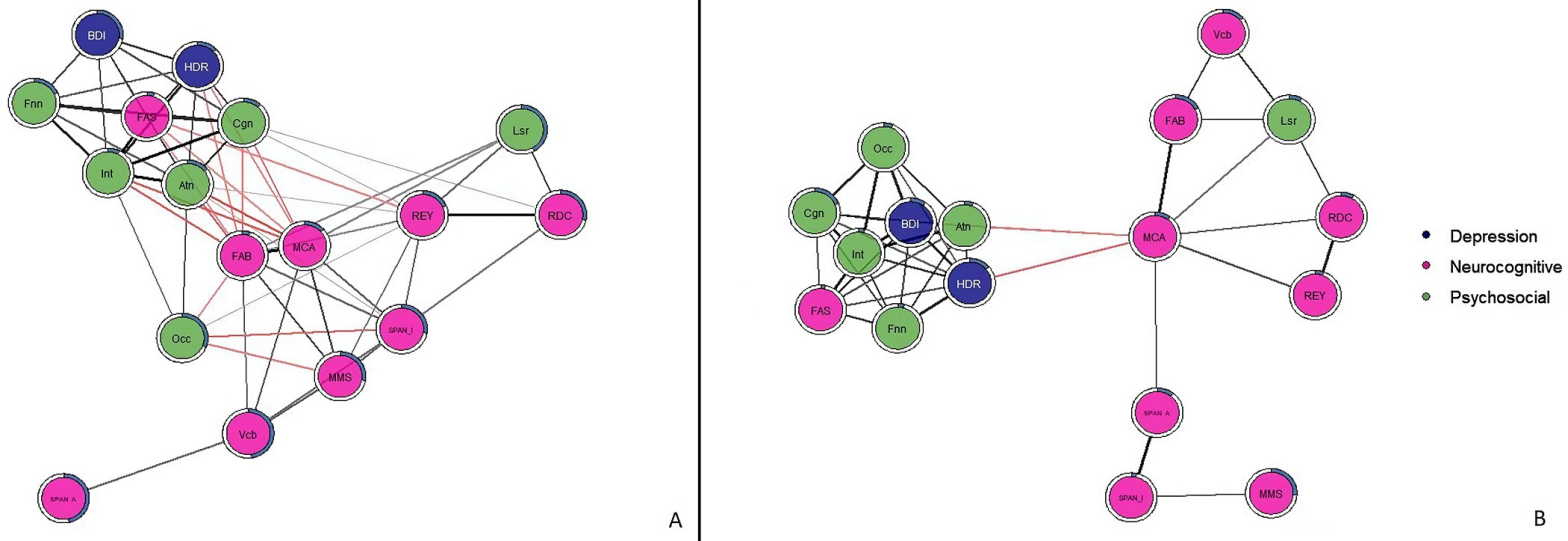
Resulting networks at first endpoint (T1). Time 1 of the study (T1). Part A represents the cluster of MDD. Part B represents the network of type I BDs. Pink color groups the neurocognitive domain, green color the psychosocial domain and blue the depression-related nodes. Black edges represent positive Spearman correlations, red edges negative Spearman correlations. The blue ring around each node represents its predictability and the fuller the bar is, the higher the RMSE value is. Cognitive domain: MCA = Montreal Cognitive Assessment, MMS = Mini Mental State Examination, FAB = Frontal Assessment Battery, FAS = Phonetic verbal Fluency, Vcb = Vocabulary, REY = Rey 15 Words immediate recall, RDC = Rey 15 Words deferred recall, SPAN_A = digit span forward, SPAN_I = digit span backward. Depression related domain (blue color in the images): BDI = Beck depression inventory II, HDR = Hamilton depression rating scale. Psychosocial domain (green color in the images): Cgn = Cognitive functioning, Occ = occupational functioning, Atn = Autonomy, Lsr = Leisure time, Int = interpersonal relationships, Fnn = financial issues.

**Fig 4:**
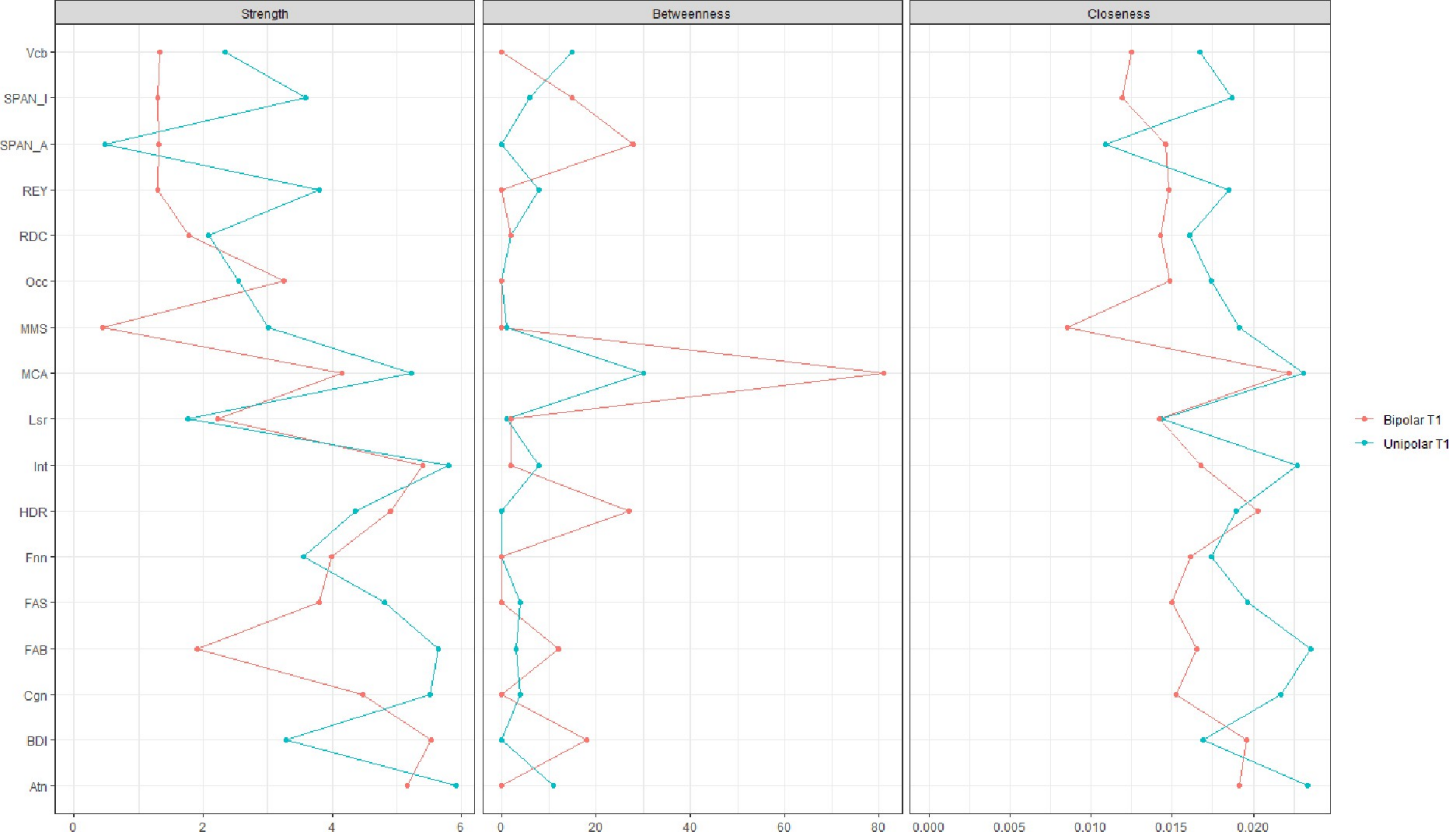
Centrality measures (strength, betweenness, closeness) of MDD and BD patients at first endpoint (T1). Red color represents BD and Blue Color MDD. MCA = Montreal Cognitive Assessment, MMS = Mini Mental State Examination, FAB = Frontal Assessment Battery, FAS = Phonetic verbal Fluency, Vcb = Vocabulary, REY = Rey 15 Words immediate recall, RDC = Rey 15 Words deferred recall, SPAN_A = digit span forward, SPAN_I = digit span backward, BDI = Beck depression inventory II, HDR = Hamilton depression rating scale, Cgn = Cognitive functioning, Occ = occupational functioning, Atn = Autonomy, Lsr = Leisure time, Int = interpersonal relationships, Fnn = financial issues.

At Time 1, after twelve weeks of treatment, a dynamic change was observed in the networks of the two groups of patients. The network of MDD patients displayed a rather different structure from the onset of the study. As for T0, at T1, the same three separate clusters of psychosocial, affective, and neurocognitive domains were observed (Figs 3A and 4). However, at T1, the neurocognitive cluster (the ensemble of nodes that represent entirely or in part the executive functions, assessed by SPAN-A; SPAN-I; REY; FAS; MoCA, MMSE, and FAB), although less connected internally, was more centrally positioned, with executive functions related to the frontal cortex driving the network and providing a link to the other two domains. More precisely, executive functions seem to drive the neurocognitive domain because they have the highest values of centrality measures. In particular the “Strength” value is indicative because it indicates how many connections start from that node. These executive function nodes provide a link to the psychosocial and affective domains because they are the only nodes to have “inter-domain” connections (neurocognitive to psychosocial and neurocognitive to affective.

Similarly, in patients with BD, twelve weeks of treatment resulted in a shift in the centrality of symptoms. In the new post-treatment scenario, depression-related indexes were more centrally placed in the network of BD. From T0 to T1, both the BDI and HDRS nodes assume a bridge function connecting the psychosocial domain and the neurocognitive domain. Moreover, the neurocognitive cluster displayed a significant loss in connectivity among its nodes (Figs 3B and 4). This indicates that from the high number of connections (correlations) among the nodes at T0, very few were conserved at T1, both within the cluster itself and among the two clusters. Thus, the network of patients with BD is less influential and, therefore, more resilient to factors that may cause it to change.

A dynamic pattern of change emerged in the network of BD with respect to what were the driving forces of the network before and after treatment. A large positive change was observed from time 0 to time 1, especially with respect to neurocognitive functions related to the frontal cortex. At time 1, these functions are more central in the communication within the network, reciprocally connecting many nodes (symptoms). Little or no change was observed in depression related parameters. In BD functions tested by the MMSE and MoCA were central in the network at time 0. AT T1 MoCA assumes a primary role connecting the neurocognitive cluster of nodes with the psychosocial cluster indicating that MoCA is the only node that has connections that link the two clusters.

In both groups MDD and BD, the predictability values of each node decreased at T1 with respect to T0. In particular, the Predictability (calculated with the RMSE: root mean squared error) Mean in MDD was 0.51 at T0 and 0.22 at T1; in BD, values diminished from 0.41 at T0 to 0.05 at T1.

The external inclusion of a “drug treatment variable”, not reported within the network but having a primary impact on network reorganization, seems to be the reason why nodes can no longer account for the influence they had on each other at T0.

In sum, in MDD patients, pharmacological treatment primarily affected executive functions which seem to improve with treatment and drive the network. In contrast, in patients with BD, twelve weeks of treatment resulted in an improvement in the overall connectivity and centrality of the affective domain, which seems then to affect and direct the network. More specifically an improvement in Connectivity was observed in relation to the number of edges connected from one-time point to the other which is further supported by the increasing values of “Strength” and “Betweenness”. In particular, the “Affective” Nodes (HDRS and BDI) show an increase in these two measures. This suggests that at T1 the two nodes have a greater impact in redistributing information in the network than they did before and that the connections with the other nodes are much stronger. In fact, Figs 2 and 3B show that there is a change in the scores of these two centrality measures (red line represents Bipolar Disorder).

## Discussion

The results of the study highlight clinically relevant differences between unipolar depression and bipolar depression at T0 and after 12 weeks of pharmacological treatment (T1). In both unipolar depression and bipolar disorder, the data indicate that treatment should be more focused on cognitive symptoms, as improvement in this domain appears to be crucial for better results in other domains, and of outcome overall.

Patients with MDD, displayed strong connectivity between psychosocial function and the affective domain while no direct connectivity with the neurocognitive domain was observed. The two realms of function were connected by the central domain of cognitive-affective evaluation, which acts as a bridge between the psychosocial domain and the neurocognitive domain. No direct connection was observed between the affective and cognitive realm of function. Our data agree with evidence coming from many recent studies suggesting that cognitive dysfunction represents a distinct biological and clinical dimension in MDD, independent from affective symptoms [3, 20, 32, 42].

Furthermore, psychosocial competence and depressive symptoms appear to form a separate world in patients with MDD, while cognitive functions with frontal affinity constitute another neighborhood/world.

The network of patients with BD, on the other hand, was characterized by a cluster of highly interconnected psychosocial nodes but guided by neurocognitive functions. However, these functions were less correlated to each other. Noteworthy, in both MDD and BD, the indices related to the affective domain were less connected and placed at the periphery of the networks which suggests that they do not play a central role.

The results of our study concur with other studies using the same methodology. Weintraub et al. (2020) [43], for example, carried out a network analysis in adolescents with bipolar disorder, which highlights the prominent role played by fatigue, depression, mood lability and irritability in the clinical symptomatology. Moreover, data presented by Chavez-Baldini et al. (2021) [44], are in line with our results, and demonstrate the crucial influence of cognition on psychopathology, cognitive functioning seems to be an independent dimension related to psychiatric clusters which interact in a transdiagnostic manner. Besides, the results from our study add a longitudinal aspect to the evidence already available: indeed, at time 1, twelve weeks after starting treatment, a dynamic change was observed in the networks of the two patient groups.

As for patients with MDD, the networks of patients with bipolar disorder display a division between the affective and cognitive domains of function, with the latter being more central. Previously, Vieta and colleagues [45] showed that euthymic bipolar patients, even after drug treatment, displayed significant impairments in executive functions [46]. These results are in accordance with a study by Godard and colleagues (2012) [47]: after a follow-up of 12 months, both unipolar and bipolar patients presented significant impairment in the cognitive realm of function, especially in executive functions (Godard et al., 2012) [47]. Moreover, Galimberti and colleagues (2020) [15], suggested that the network of patients with bipolar depression display greater executive dysfunction than that of patients with MDD. Data from Kapczinski (2016) [1] are also aligned with these results. As in bipolar patients, severe depression has been associated with lower scores in the domain of executive functioning, examined using the FAST [1].

In addition, in patients with bipolar disorder, connections were stronger within the cognitive domain. Therefore, we can assert that for these patients, pharmacological treatment is more effective in producing a change in cognitive functioning than for patients with MDD. This is coherent with the literature sustaining that treating cognitive symptoms is a critical step in the clinical approach to depression, and will help to improve psychosocial functioning, enhance the quality of life and avoid relapses [25, 33, 48].

The differences observed in network structure and connections lead to think that in MDD, the psychosocial area is primarily affected. The increase in severity of symptoms results in rapidly enhanced impairment in various areas of social life and autonomy of the individual because of the high density of connections. In turn, this leads to decreased performance on the global cognitive scales (negative connections between the two clusters) which, then, diminish the performance in the remaining cognitive nodes. As a result, patients lack the mental capacity to cope with the situation they find themselves in.

**Fig 5:**
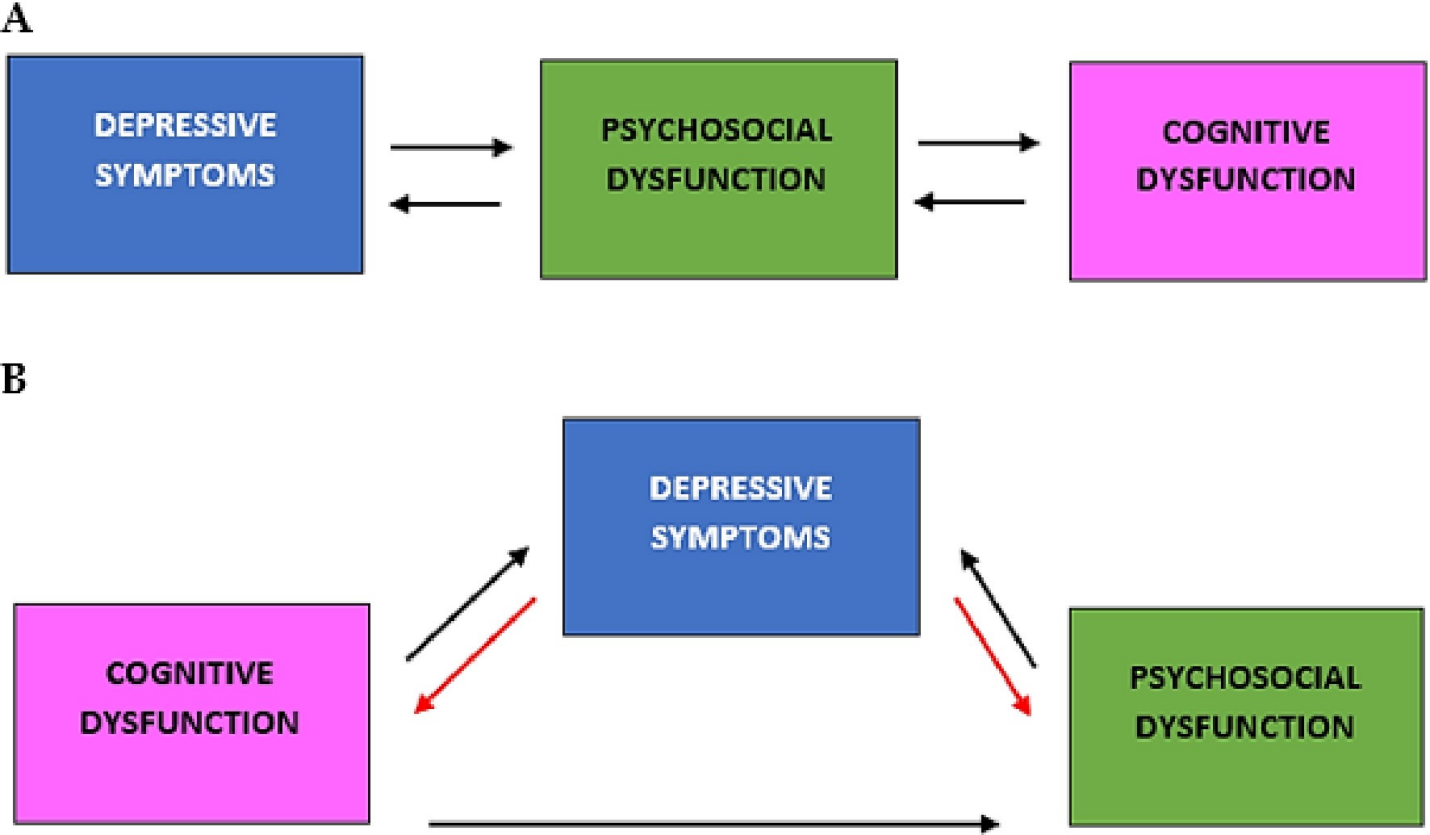
Domains and their possible interaction in MDD and BD. Part A: Possible interactions among domains for MDD by analyzing patients’ test performance and resulting networks. Part B: Possible interactions among domains for BD by analyzing patients’ test performance and resulting networks. Color coding is consistent with network images. Black arrows represent the main path of interactions between studied functions. Red arrows should be interpreted as a secondary effect due to black arrows.

Given that the connection between each pair of nodes in the network is not directed, and therefore not absolute, a mechanism is triggered that self-feeds the symptoms which leads patients to experience extreme difficulty in finding alternative ways to cope with the situation (Fig 5A). In addition, a hyperconnected network will take much longer to stabilize after the trigger has disappeared which might lead to or favor chronicity or recurrence of depressive episodes.

The predictability index gives additional information about the structure of the network at T0. The results show that the valence loops are on average full in MDD. This suggests that much of the variance of those variables is explained by the neighboring nodes with which they have direct connections. At the qualitative level, predictability introduces the possibility to consider change by working both on the variable itself as well as on neighboring nodes. Predictability, together with hyper-connectivity, suggests that in MDD, the psychosocial, neurocognitive, and affective variables influence each other and, to a large extent, explain their variability. Consequently, treating the depressive symptomatology and providing cognitive strategies at the same time is likely the most appropriate way to cope with this pathology.

The structure that emerges from the analysis of BD networks displays a different pattern. A breakdown of cognitive aspects, especially frontal ones, is evident from an early stage, and does not seem to have a direct impact on depressive symptoms and psychosocial impairment. In addition, agitation, manic episodes, irritability, insomnia, and other specific symptoms of bipolar disorder primarily involved the psychosocial sphere which represents the weaker cluster (greater number of connections). Combining these two aspects, the data suggest e that cognitive dysfunction and psychosocial dysfunction both contribute to the development and worsening of depressive symptoms. In turn, this may centrally direct and increase psychosocial impairment (Fig 5B). In contrast, frontal functions lack a primary role in the network and are unable to control or mitigate depressive symptoms.

Finally, predictability proved to be an important additional parameter to diversify the dynamics within a network and provided new insights for differential diagnosis at baseline. RMSE values, on average, were lower in BD than in MDD.

Therefore, we can postulate that in MDD the variability of each node is better explained among the nodes/variables analyzed. On the contrary, values are lower in networks of patients with BD. This may be attributed to the fact that intrinsic factors, such as, genetic predisposition play a more significant role in BD than in MDD. Also, the patients in this study all had rather severe forms of both disorders and were often hospitalized during the treatment period. Creating networks including predictability might be even more important for patients with less severe symptomatology. Here, prediction of risk might allow us to work preventively and consider the strengths and needs resulting from the patients’ network to develop preventive treatment strategies, among which cognitive therapy.

Taken together, our findings suggest that treatment of MDD should include tailored cognitive therapy, because improvement in this central domain appears to be fundamental for better outcomes in other domains. Likewise, in BD, treatment should include both pharmacological and non-pharmacological interventions, which, in turn, may lead to possible improvements in the other domains due to their pivotal role in driving change.

## Conclusions

As suggested from recent studies [49], a dynamic network approach represents a novel tool to identify distinct patterns of interacting symptoms in neuropsychiatric disorders. The dynamic network approach that combines the relationships between risk and protective factors from different realms of functions has the advantage that it is:

1. Personalized: it offers insight in therapy options focused on the clinical trajectory of individual patients with MDD or BD. In addition, it provides alternative targets to take into consideration when making treatment and follow-up care decisions.
2. Collaborative: it engages the patient in the process of care offering individual guidance regarding their strengths and needs.
3. Efficient: assist in finding the best treatment for the patient and reach the right balance based on an integrated process involving different fields of function.
4. Predictive: predicts the trajectory of future disease manifestations of patients diagnosed with MDD or BD and assists in defining risk while offering a highly useful approach in planning and surveilling a combination of pharmacological treatment and psychotherapy.

In conclusion, our network analysis established unique patterns of interconnected domains of function and for each disorder this “depressive disorder connectome” will help to recognize interrelated behaviors allowing to isolate the domain(s) most central to the overall risk and distinguish different trajectories thus improving successful programs fundamental for the surveillance and monitoring of personalized interventions.
